# Anaesthetic Management of Renal and Liver Transplantation Recipients During Caesarean Section

**DOI:** 10.5152/TJAR.2023.22033

**Published:** 2023-04-01

**Authors:** Aynur Camkıran Fırat, Asude Ayhan, Coşkun Araz, Nükhet Akovalı, Zeynep Kayhan

**Affiliations:** 1 Departments of Anaesthesiology, Başkent University Faculty of Medicine, Ankara, Turkey; 2 Departments of General Surgery, Başkent University Faculty of Medicine, Ankara, Turkey

**Keywords:** Cesarean section, general anaesthesia, liver transplantation, regional anaesthesia

## Abstract

**Objective:**

The aim of this study was to present our experience in liver transplantation recipients and renal transplantation recipients during caesarean section.

**Methods:**

Retrospective data regarding liver transplantation recipients and renal transplantation recipients who underwent caesarean section between January 1997 and January 2017 have been collected from the hospital records.

**Results:**

Fourteen live births occurred from 5 liver transplantation recipients and 9 renal transplantation recipients, all of them from caesarean section. The mean maternal age (28.4 ± 4.0 years vs. 29.2 ± 4.1 years, *P* = .38), body weight before conception (57.4 ± 8.8 kg vs. 64.5 ± 8.2 kg, *P* = .48), and the time from transplantation to conception (99.0 ± 50.7 months vs. 101.0 ± 57.5 months, *P* = .46) were similar for 5 liver transplantation recipients and 9 renal transplantation recipients, respectively. Four caesarean sections were performed under general anaesthesia, whereas spinal anaesthesia was used in 10 patients. The mean birth weight was similar (2502 ± 311g vs. 2161 ± 658 g, *P* = .3). There were 3 premature deliveries in liver transplantation recipients versus 6 premature deliveries in renal transplantation recipients and 2 low-birth-weight infants (<2500 g) in liver transplantation recipients versus 4 in renal transplantation recipients among 14 newborns. Infants small for gestational age were diagnosed in 9/14 (3 liver transplantation recipients versus 6 renal transplantation recipients, *P* = 1).

**Conclusion:**

General and regional anaesthesia can be safely used during caesarean delivery of liver transplantation recipients and renal transplantation recipients without increased risk of graft losses. Prematurity and low birth weight were mainly due to the cytotoxic drugs for immunosuppression. There are no differences in liver transplantation recipients and renal transplantation recipients for maternal and foetal complications according to our data.

**Main Points**

- General and regional anaesthesia can be safely used during caesarean section of the liver transplantation recipients (LTRs) and renal transplantation recipients (RTRs) without increased risk of graft losses.
- Prematurity and low birth weight were mainly due to the cytotoxic drugs for immunosuppression.
- There are no differences in LTRs and RTRs for maternal and foetal complications according to our data.
- In addition, these pregnancies must be followed by a multidisciplinary approach.
- General and regional anaesthesia had a similar safety profile and can be applied according to the patient’s needs.

## Introduction

Successful pregnancy after solid organ transplantation is possible but with the risks of some obstetrical problems such as hypertension, graft rejection, infection, preeclampsia, preterm birth, intrauterine growth retardation, congenital malformations, intrauterine death, spontaneous abortion, and low birth weight.^1^ Also, the process is difficult to manage for transplantation physicians, obstetricians, and anaesthesiologists.^2^ Even if allograft organ function is perfect, the pregnancies have several risks, and there are lots of risks for the mother, for the transplanted organ, and especially for the baby. There are no prospective, randomized controlled trials conducted on these groups of patients.

Both general and spinal anaesthesia are usually used for caesarean section (C/S); in addition, both of them have a similar safety profile. Spinal anaesthesia has some advantages such as the mother is awake, the drug dose is small, minimal depression of the neonate, and avoidance of risk of endotracheal intubation, but the disadvantages include hypotension, bradycardia, and postdural puncture headache. Many anaesthesiologists defend that hypotension may be treated by fluid boluses. Afolabi and Lesi^3^ showed that there is no evidence that spinal anaesthesia is superior to general anaesthesia, and they also reported that future researches may describe neonate morbidity and mother outcomes. An important issue in obstetric anaesthesia is spinal hypotension during C/S. Prevention of spinal hypotension may be possible, but the ideal method has not been defined.

The aim of this study was to present our experience in liver transplantation recipients (LTRs) and renal transplantation recipients (RTRs) during C/S. In addition, we analysed the effects of pregnancy and delivery on the function of the transplanted organ.

## Methods

After obtaining approval from the Institutional Review Board, retrospective data regarding LTRs and RTRs who underwent C/S at Başkent University Hospital in Ankara between January 1997 and January 2017 have been collected from the hospital records. Perioperative data related to anaesthetic management and intraoperative events were collected along with the information related to postoperative course and survival to hospital discharge.

Retrospective data regarding LTRs and RTRs who underwent C/S among 618 liver transplantations and 2803 renal transplantations at Başkent University Hospital in Ankara between January 1977 and December 2017 have been collected from the hospital records. Among these recipients, 210 LTRs and 382 RTRs consisted of women of childbearing age or girls who may become pregnant later in life. We evaluated 14 deliveries, all of which were first deliveries. There were no twins or triplets. All of them were C/S, and we did not evaluate vaginal deliveries.

The information gathered from the subjects’ records included demographic features of gender, age, and weight, comorbidities, perioperative laboratory values, use and volume of packed red blood cells, fresh-frozen plasma, platelets, vasopressors, anaesthesia duration, and urine output. We also noted the length of stay in hospital as well as the mortality rates.

### Statistical Analysis

All data were analysed with Statistical Package for the Social Sciences (SPSS) software (version 20.0, IBM SSPS Corp.,., Armonk, NY, USA). The 2 groups were compared using the Chi-square and Mann–Whitney *U*-tests when appropriate. The data are expressed as mean values ± SD. *P* < .05 was considered significant in this study.

## Results

Fourteen live births occurred from 5 LTRs and 9 RTRs, and all of them were C/S. No women gave birth twice during this period. Patient data are presented in Table 1. The mean maternal age (28.4 ± 4.0 years vs. 29.2 ± 4.1 years, *P* = .38) and body weight before conception (57.4 ± 8.8 kg vs. 64.5 ± 8.2 kg, *P* = .48) did not differ between LTRs and RTRs. The time from transplantation to conception (99.0 ± 50.7 months vs. 101.0 ± 57.5 months, *P* = .46) and the rate of primipara were similar for LTRs and RTRs, respectively.

All recipients were maintained on cyclosporine, azathioprine, tacrolimus, and corticosteroids before and during pregnancy for immunosuppression. Four C/Ss were performed under general anaesthesia (1 LTRs vs. 3 RTRs, *P* > .05), whereas spinal anaesthesia was used in 10 patients (Table 2). Liver and renal function tests were stable in all of the patients, and we did not observe any acute or subacute rejection (Table 3). After delivery, RTRs did not have deterioration in renal function. Only 1 mother with renal transplantation died in 1 year after delivery. In both the groups, the aspartate transaminase, the alanine transaminase, direct bilirubin, creatinine, and blood urea nitrogen levels were analysed first 48 hours after operation for graft function.

The gestational age at birth was similar (36.2 ± 1.7 weeks vs. 35.1 ± 2.8 weeks, *P* = .47) (Table 1). The mean birth weight was similar (2502 ± 311g vs. 2161 ± 658 g, *P* = .3) (Table 4). Infants small for gestational age were diagnosed in 9/14 (3 LTRs vs. 6 RTRs, *P* = 1). We recorded 9 premature deliveries (3 premature deliveries in LTRs and 6 premature deliveries in RTRs) before 37 weeks of gestation. Of these, 4 premature deliveries were before 35 weeks of gestation (1 of them in LTRs and 3 of them in RTRs) (Table 4). There were 2 low-birth-weight infants (<2500 g) in LTRs vs. 4 in RTRs among 14 newborns. Four of them (1 in LTRs vs. 3 in RTRs, *P* = .65) were very low-birth-weight infants (<2000 g) (Table 4). None of the neonates died. One of them in RTRs and very low birth weight was with Goldenhar syndrome it is craniofacial syndrome with cardiac abnormalities. The total appearance, pulse, grimace, activity, and respiration (APGAR) scores at 1 minute (8.2 ± 0.8 vs. 7.1 ± 1.7, *P* = .49) and 10 minutes (9.4 ± 0.5 vs. 9.0 ± 0.5, *P* = .37) were similar. The APGAR score of a baby who was urgently taken by C/S under general anaesthesia was 3 in the first minute. All other babies had APGAR scores of 7 and above at both the first and fifth minutes. Mother and neonate hospitalization durations were not different.

## Discussion

This article evaluated anaesthesia management in LTRs and RTRs during C/S. Moreover, the effects of pregnancy and delivery on the transplanted organ function were assessed. The results of this study suggested that there were 9 pregnancies with kidney grafts and 5 pregnancies with liver grafts. All patients were primipara, and neither of them were multiple pregnancies. The graft functions were not affected at least for the first 48 hours after deliveries.

Blume et al^2^ worked on the same issue, and they especially emphasized the optimal timing from transplantation to conception. They reported that the time must be longer than 1 year because of graft function and hemodynamic stability. However, the status of stable graft function on reduced immunosuppression, stable blood pressure and kidney function, and the absence of infectious complications should be provided 1-2 years after the transplantation according to The American Society of Transplantation Consensus Group.^4^ But, the consensus brief has not been updated from 2005. In Moaveni et al’s^5^ review, they revised the good time for timing of pregnancy after transplantation and highlighted 4 conditions: no episodes of rejection in the past year, a low risk for opportunistic infections, stable renal function, and a low dose of maintenance immunosuppression. The duration between transplantation and conception for our patients was longer than 2 years, and all graft functions remained stable after delivery. Our patients did not need any haemodialysis or plasmapheresis. Moaveni et al^5^ also reported the rate of caesarean deliveries at their center and found out that it’s prevalence was higher than that reported in the existing literature. We analysed only caesarean deliveries in this article. We think that preterm labour, small gestational age, and other obstetrical problems affect obstetricians and anaesthesiologists to stay in safe and hesitate about delivery and anaesthesia methods.

Our data showed that pregnancy after RTRs and LTRs does not appear to increase the risk of graft loss for neither liver nor kidney. Songin et al^6^ analysed graft function and pregnancy survival after solid organ transplantation. In addition, they compared their data between RTRs and LTRs. On the other hand, Zeyneloglu et al^7^ evaluated only RTRs. Solid organ transplantation and perioperative management of RTRs should be handled by a team including anaesthesiologists. Moaveni et al^5^ informed that the team must decide the type of delivery and timing of delivery and been supported optimal anaesthesia management for each individual pregnant woman by anaesthesiologist. The team must know and understand transplanted organ physiology and side effects related to immunosuppressive agents.^5,8^

The most important problem is preterm delivery in LTRs and RTRs during pregnancy.^9^ We recorded 9 premature deliveries (3 premature deliveries in LTRs and 6 premature deliveries in RTRs). Four of them were before 35 weeks of gestation (1 of them in LTRs and 3 of them in RTRs). Statistical analysis did not show any significant effects between LTRs and RTRs in our patients. But Songin et al^6^ said that preterm delivery is important especially in patients after renal transplantation (74.4% in the RTR group vs. 43.75% in the LTR group). Our results do not support the idea, and the reason for this may be our patient size. We evaluated only 14 pregnancies in women after solid organ transplantation. All newborn babies were delivered in good general condition: the total APGAR score at 5 minutes after birth ranged from 7 to 10. The 9 neonates (60% in the LTR group vs. 66% in the RTR group) born before 37 weeks of gestation presented characteristics of prematurity. Six of 14 neonates (20% in the LTR group vs. 33.3% in the RTR group) demonstrated low birth weights, which were defined as less than 2500 g.

Both regional and general anaesthesia techniques are currently being used for renal transplantation. It is not clear whether one of these is superior to other in terms of intraoperative complications.^5^ Our 4 C/Ss were performed under general anaesthesia (1 LTRs vs. 3 RTRs, *P* > .05), whereas spinal anaesthesia was used in 10 patients. Intraoperative vasopressor requirements were similar for both anaesthesia techniques. No intraoperative complications were noted.

The limitation of this study is its retrospective nature. There are many factors facilitating (type of graft, comorbidity, maintance immunosuppression, etc) the transplantation in patients with perioperative. Considering all these factors, we may have not standardized the 2 groups completely. However, we did not evaluate the causes of organ transplantation.

## Conclusion

In summary, general and regional anaesthesia can be safely used during caesarean delivery of the LTRs and RTRs without increased risk of graft losses. Prematurity and low birth weight were mainly due to the cytotoxic drugs for immunosuppression. There are no differences in LTRs and RTRs for maternal and foetal complications according to our data. In addition, these pregnancies must be followed by a multidisciplinary approach. General and regional anaesthesia had a similar safety profile and can be applied according to the patient’s need.

## Figures and Tables

**Table 1.** Characteristic of the Patients (n = 14)

Variables	Liver Transplant Recipients (n = 5)	Renal Transplant Recipients (n = 9)	*P*
Age (years)	28.4 ± 4.0	29.2 ± 4.1	.38
Body weight (kg)	57.4 ± 8.8	64.5 ± 8.2	.48
Primipara	5 (100)	9 (100)	1
Length of gestational age (weeks)	36.2 ± 1.7	35.1 ± 2.8	.47
Time from transplantation to conception (months)	99.0 ± 50.7	101.0 ± 57.5	.46

Values are expressed as mean ± SD or number (%).

**Table 2.** Intraoperative Management (n = 14)

Variables	Liver Transplant Recipients (n = 5)	Renal Transplant Recipients (n = 9)	*P*
General anaesthesia	1 (20)	3 (33.3)	1
Emergency operation	2 (40)	6 (66.6)	.58
Extubation in operating room	1 (20)	3 (33.3)	1
Vasopressors (ephedrine, mg)	2.0 ± 4.4	2.2 ± 3.6	.92
Requirement of ICU	1 (20)	2 (22.2)	.75

Values are expressed as mean ± SD or number (%).

ICU, intensive care unit.

**Table 3.** Laboratory Values (n = 14)

Variables	Liver Transplant Recipients (n = 5)	Renal Transplant Recipients (n = 9)
**Preoperative laboratory values**		
Haemoglobin (g dL^-1^)	11.3 ± 1.7	10.3 ± 0.7
Blood urea nitrogen (mg dL^-1^)	6.9 ± 1.5	18.8 ± 9.20
Creatinine (mg dL^-1^)	0.6 ± 0.1	1.7 ± 1.6
Aspartate aminotransferase (U L^-1^)	17.8 ± 10.3	14.5 ± 2.9
Alanine transaminase (U L^-1^)	15.8 ± 11.9	7.8 ± 2.2
Direct bilirubin (mg dL^-1^)	0.4 ± 0.3	0.3 ± 0.1
**Postoperative day 1**		
Haemoglobin (g dL^-1^)	8.9 ± 1.2	9.2 ± 1.2
Blood urea nitrogen (mg dL^-1^)	8.2 ± 1.9	19.6 ± 9.7
Creatinine (mg dL^-1^)	0.6 ± 0.2	1.9 ± 2.6
Aspartate aminotransferase (U L^-1^)	20.4 ± 7.8	15.9 ± 3.5
Alanine transaminase (U L^-1^)	12.4 ± 6.3	8.0 ± 2.8
Direct bilirubin (mg dL^-1^)	0.5 ± 0.3	0.4 ± 0.1
**Postoperative day 2**		
Haemoglobin (g dL^-1^)	9.4 ± 0.8	9.3 ± 0.6
Blood urea nitrogen (mg dL^-1^)	8.2 ± 2.4	20.4 ± 7.4
Creatinine (mg dL^-1^)	0.6 ± 0.1	1.5 ± 1.4 ± 6.3
Aspartate aminotransferase (U L^-1^)	19.4 ± 10.3	15.8 ± 6.3
Alanine transaminase (U L^-1^)	12.5 ± 6.9	7.3 ± 2.9
Direct bilirubin (mg dL^-1^)	0.4 ± 0.2	0.3 ± 0.1

Values are expressed as mean ± SD.

**Table 4.** Neonatal Status (n = 14)

Variables	Liver Transplant Recipients (n = 5)	Renal Transplant Recipients (n = 9)	*P*
Preterm deliveries (<37 weeks)	3 (60)	6 (66.6)	.80
Preterm deliveries (<35 weeks)	1 (20)	3 (33.3)	.63
Neonatal weights, g	2502.0 ± 311.8	2161.1 ± 658	.30
Neonatal weight < 2500 g	2 (40)	4 (44.4)	.65
Neonatal weight < 2000 g	1 (20)	3 (33.3)	.65
APGAR score at 1 minute <7	0	1	.49
APGAR score at 5 minutes <7	0	0	.37

Values are expressed as mean ± SD or number (%).

APGAR score, appearance, pulse, grimace, activity, and respiration.
